# Computer-guided discovery of epigenetics drugs: molecular modeling and identification of inhibitors of DNMT1

**DOI:** 10.1186/1758-2946-4-S1-P25

**Published:** 2012-05-01

**Authors:** Jakyung Yoo, José L Medina-Franco

**Affiliations:** 1 Torrey Pines Institute for Molecular Studies, Port St. Lucie, Florida, 34987, USA

## 

DNA methylation is a covalent chemical modification of DNA catalyzed by DNA methyltransferases (DNMTs) and plays a crucial role in epigenetic modifications. Inhibition of DNMT is a promising strategy for the treatment of various developmental and proliferative diseases, particularly cancers. Molecular docking and other computational approaches are increasingly being used to explore the ligand-binding interactions of DNMT inhibitors [1,2]. In this work we conducted molecular docking of experimentally known active compounds in the catalytic site of the recently published crystal structure of DNMT1 [3]. Prior docking, the conformation of the catalytic site was modelled with molecular dynamics into an active conformation. To our knowledge, this is the first molecular modelling study conducted with the catalytic binding site of this crystal structure. Based on the docking results, we developed a structure-based pharmacophore model. Molecular modelling results were compared with the insights previously obtained with a homology model of the methyltransferase domain of DNMT1 [4]. We also discuss the experimental inhibitory activity and docking of a novel DNMT1 inhibitor recently identified in our group [5]. Results of this work have direct implications in the future computer-based screening and optimization of inhibitors of DNMT1 and show that computational approaches form part of multidisciplinary efforts to further advance epigenetic therapies [1].
